# Community-acquired pneumonia in hospitalised patients: changes in aetiology, clinical presentation, and severity outcomes in a 10-year period

**DOI:** 10.1080/07853890.2022.2138529

**Published:** 2022-11-04

**Authors:** Júlia Sellarès-Nadal, Joaquín Burgos, María Teresa Martín-Gómez, Andrés Antón, Roger Sordé, Daniel Romero-Herrero, Pau Bosch-Nicolau, Anna Falcó-Roget, Cristina Kirkegaard, Dolors Rodríguez-Pardo, Oscar Len, Vicenç Falcó

**Affiliations:** a Departament de Medicina, Universitat Autònoma de Barcelona, Bellaterra, Spain; b Infectious Diseases Department, Vall d’Hebron Barcelona Hospital Campus, Vall d’Hebron Hospital Universitari, Barcelona, Spain; c Malalties Infeccioses Vall d’Hebron Institut de Recerca (VHIR), Vall d’Hebron Barcelona Hospital Campus, Vall d’Hebron Hospital Universitari, Barcelona, Spain; d Microbiology Department, Vall d’Hebron Barcelona Hospital Campus, Vall d’Hebron Hospital Universitari, Barcelona, Spain; e Internal Medicine Department, Hospital de Trauma Manuel Giagni, Ministerio de Salud Pública y Bienestar Social, Asunción, Paraguay

**Keywords:** Pneumonia, virology, *Staphylococcus aureus*, MRSA, mortality

## Abstract

**Background and objective:**

Community-acquired pneumonia (CAP) is a frequent cause of hospitalisation. Several factors, such as pandemics, vaccines and globalisation may lead to changes in epidemiology, clinical presentation, and outcomes of CAP, which oblige to a constant actualisation. We performed this study to analyse how these factors have evolved over a 10-year period.

**Materials and methods:**

Patients diagnosed with CAP for two 1-year periods that were 10 years apart (2007–2008 and 2017–2018) were included. We compared microbiological information, clinical data and evolutive outcomes in the two periods. A mortality analysis was performed.

**Results:**

1043 patients were included: 452 during the first period (2007- 2008), and 591 during the second period (2017–2018). Bacterial aetiology did not change during the 10-year period, besides a slight increase in *Staphylococcus aureus* (0.9% vs 2.9%, *p* = 0.026). There was a decline in the proportion of bacteraemia in the second period (14.8% vs 9.6%, *p* = 0.012). The incidence of complicated pleural effusion and septic shock declined too (6.4% vs 3.6%, *p* = 0.04 and 15.5% vs 6.3%, *p* < 0.001). Respiratory failure and Intensive care unit (ICU) admission were similar in both periods. Variables independently associated with mortality were age and septic shock. Influenza vaccine was a protective factor against mortality in the second period.

**Conclusions:**

We have not found relevant differences in the bacterial aetiology of CAP over this 10-year period. There has been a decline in septic complications of CAP such as septic shock, bacteraemia, and complicated pleural effusion. Influenza vaccination is an important tool to reduce mortality.

**KEY MESSAGES**
- There were no differences in the bacterial pathogens causing CAP among the 10-year study period. There has been a decline in septic complications of CAP such as septic shock, bacteraemia, and complicated pleural effusion.

## Introduction

Community-acquired pneumonia (CAP) is one of the main challenges in infectious diseases, as it represents an important cause of morbidity and mortality, and it is a frequent cause of hospitalisation worldwide [1–3]. Moreover, infectious diseases are a dynamic field, in which epidemiology is constantly evolving. Changes in environmental factors, the introduction of new vaccines and globalisation itself may lead to changes in epidemiology, clinical presentation, and severity of CAP. Therefore, periodic survey studies are needed to ensure that clinical guidelines remain adequate [4,5].

With the development of new diagnostic methods such as molecular testing, there has been an improvement in the identification of etiologic causes of CAP [6,7]. This has led to an increase of viral detection in patients with CAP, reaching up to 24.5–30% of all cases [8,9]. Some bacteria, such as *Staphylococcus aureus,* have traditionally been related to viral co-infection. Recent guidelines recommend the investigation of respiratory viruses mainly during the flu-season [4], however, the impact on bacterial aetiology and clinical outcomes of patients has been barely evaluated. With such diagnostic approach, we wonder if there has been changes in the pathogens detected and in the outcomes of patients with CAP in the last years.

Another relevant change affecting the management of respiratory-tract infections in the latest years is the expansion of vaccination programs with higher vaccination targets [10]. On the one hand, it is known that influenza vaccine reduces the rates of influenza infection as well as improves the prognosis of patients [11]. On the other hand, in our setting the 13-valent conjugate pneumococcal vaccine was introduced in 2010, and there is little information on its impact on clinical presentation and severity outcomes of adult patients with CAP [12]. We wonder if vaccination policies have had an impact on the severity and outcomes of patients with CAP in the last decade.

We designed this study to analyse if there have been changes in the epidemiology, clinical presentation, and outcomes of CAP over a 10-year period. We included patients diagnosed with CAP for two 1-year periods that were 10 years apart (2007–2008 and 2017–2018). Between these 2 periods detection of respiratory virus, mainly during the flu season, was implemented in our hospital. The objectives of the study were to study if there have been changes in the clinical presentation, bacterial aetiology, and outcomes of patients with CAP between these two periods.

## Materials and methods

### Study design and inclusion criteria

This is an observational study of all consecutive adult inpatients (age ≥18 years) diagnosed with CAP in two periods separated by 10 years: 2007–2008 and 2017–2018. The study was conducted at the Hospital Universitari Vall d’Hebron, a 1100 beds-tertiary teaching hospital in Barcelona. Patients with nosocomial pneumonia and those with evidence of aspiration pneumonia (dysphagia, altered gag reflex, low level of consciousness) were excluded.

### Data collection

We collected epidemiologic information (age, sex, residency in nursing home, smoking, alcohol consumption and vaccination status), comorbidities (hypertension, chronic obstructive pulmonary disease (COPD), diabetes mellitus, chronic renal failure, neurological disorders, and neoplasms) and immunosuppressive factors (solid organ transplantation, haematopoietic transplantation, chemotherapy, long-term use of corticosteroids, and HIV infection). We also registered clinical information, laboratory results, radiological findings, microbiological information, and severity data (septic shock and respiratory failure). Empirical treatment was recorded. Evolutive variables, such as admission at the Intensive Care Unit (ICU) and in-hospital mortality were collected. CURB-65 score and Pneumonia Severity Index (PSI) were calculated.

### Microbiologic procedures

Microbiologic diagnostic procedures were performed according to the hospital protocol and included (1) two sets of blood cultures, (2) when available, qualitative and semi-quantitative culture of a good quality sputum sample as previously defined [13], (3) in patients who required orotracheal intubation, an endotracheal aspirate or bronco-alveolar-lavage samples (4), urinary antigen for *Streptococcus pneumoniae* in all patients, (5) urinary antigen for *Legionella pneumophila* if there was clinical or epidemiological suspicion and in all cases of severe CAP. Other microbiological techniques such as PCR for *S. pneumoniae* in pleural fluid or serologic determinations for antibodies against atypical pathogens were performed according to clinical or epidemiological suspicion. We considered a positive sputum culture when the semi-quantitative culture yielded > 1.000.000 colony-forming units (CFU) or when the microorganism was found to be predominant in qualitative cultures.

The only difference in the microbiologic procedures performed in the two periods was viral detection. In the second period (2017–2018) a multiplex real-time PCR determination of respiratory virus (Influenza A and B, including Flu A-H1pdm09, Respiratory Syncytial Virus A and B, Adenovirus, Enterovirus, Metapneumovirus, Parainfluenza 1–4 Virus, Rhinovirus, Bocavirus 1–4, and Coronavirus NL63, OC43 and 229E) in a nasopharyngeal swab was performed if requested by the attending physician in case of clinical or epidemiological suspicion (Allplex™ Respiratory Panels 1, 2 and 3, Seegene Inc., Korea). During the flu season (from November 2017 to March 2018) a rapid narrow-range real-time PCR for influenza virus was also performed (Xpert Flu/RSV^→^. Cepheid, Sunnyvale, CA, USA).

### Definitions

Pneumonia was defined as the presence of signs or symptoms of respiratory-tract infection (cough, fever, purulent sputum, pleuritic chest pain or pulmonary semiology compatible with lung consolidation) associated with the presence of a newly visualised infiltrate in the chest radiography.

Bacterial pneumonia was diagnosed in patients when (1) a microorganism likely to cause bacterial pneumonia was isolated in blood, pleural fluid, acceptable-quality sputum, endotracheal aspirate or bronco-alveolar-lavage samples, (2) a PCR for *S. pneumoniae* was positive in pleural fluid, (3) a urinary antigen test for *S. pneumoniae* or *L. pneumophila* was positive, (4) seroconversion of *L. pneumophila*, *M. pneumoniae*, *C. pneumoniae*, *C. psittaci* and *C. burnetii* antibody titres was documented. Viral infection was considered in all patients presenting a positive real PCR test for respiratory virus, regardless of the detection of a bacterial microorganism. Respiratory failure was defined as pO2 in arterial blood lower or equal to 60 mmHg or peripheral pulse oximetry lower than 90%. Septic shock was defined as the need of vasopressors to maintain medium blood pressure over 65 mmHg despite an adequate volume status.

### Treatment

Patients received antibiotic treatment according to current local protocols, which did not change between the two study periods. Hospitalised patients with non-severe CAP received amoxicillin-clavulanic acid and in those with severe CAP, a third-generation cephalosporin (ceftriaxone or cefotaxime) associated with a macrolide (azithromycin) was prescribed. Fluoroquinolones were recommended in cases of penicillin allergy or as an alternative treatment at the attending physician discretion when there was a clinical suspicion for atypical pneumonia. In the second period, a 5-day course of oseltamivir was recommended for inpatients with confirmed influenza infection.

### Statistical analysis

We performed a descriptive analysis of basal characteristics, clinical and microbiological information of the study population. Categorical variables are expressed as percentages and numerical data as mean and standard deviation (SD) in cases of normal distribution or as median and interquartile range (IQR) for non-normally distributed data. Variables were compared between the two periods using the Chi-square test for qualitative variables and T-student test for quantitative variables. We performed first a multivariate analysis (forward onwards) by binary logistic regression to identify variables independently associated with mortality. Secondly, we performed a multivariate analysis of mortality in each period in order to know the evolution of risk factors in both periods. Significant (*p* < 0.05) variables from the univariate analysis were included in the multivariate analysis. Statistical analyses were performed using IBM SPSS Statistics for Windows, Version 20.0. Armonk, NY; IBM Corp. Released 2011.

### Ethics statement

The study was approved by the Ethics Committee of Vall d’Hebron Research Institute (registration code PR(AG)345/2018). Need for informed consent was waived, as data and samples were analysed retrospectively, and the study was non-interventional in nature.

## Results

**Figure 1.**
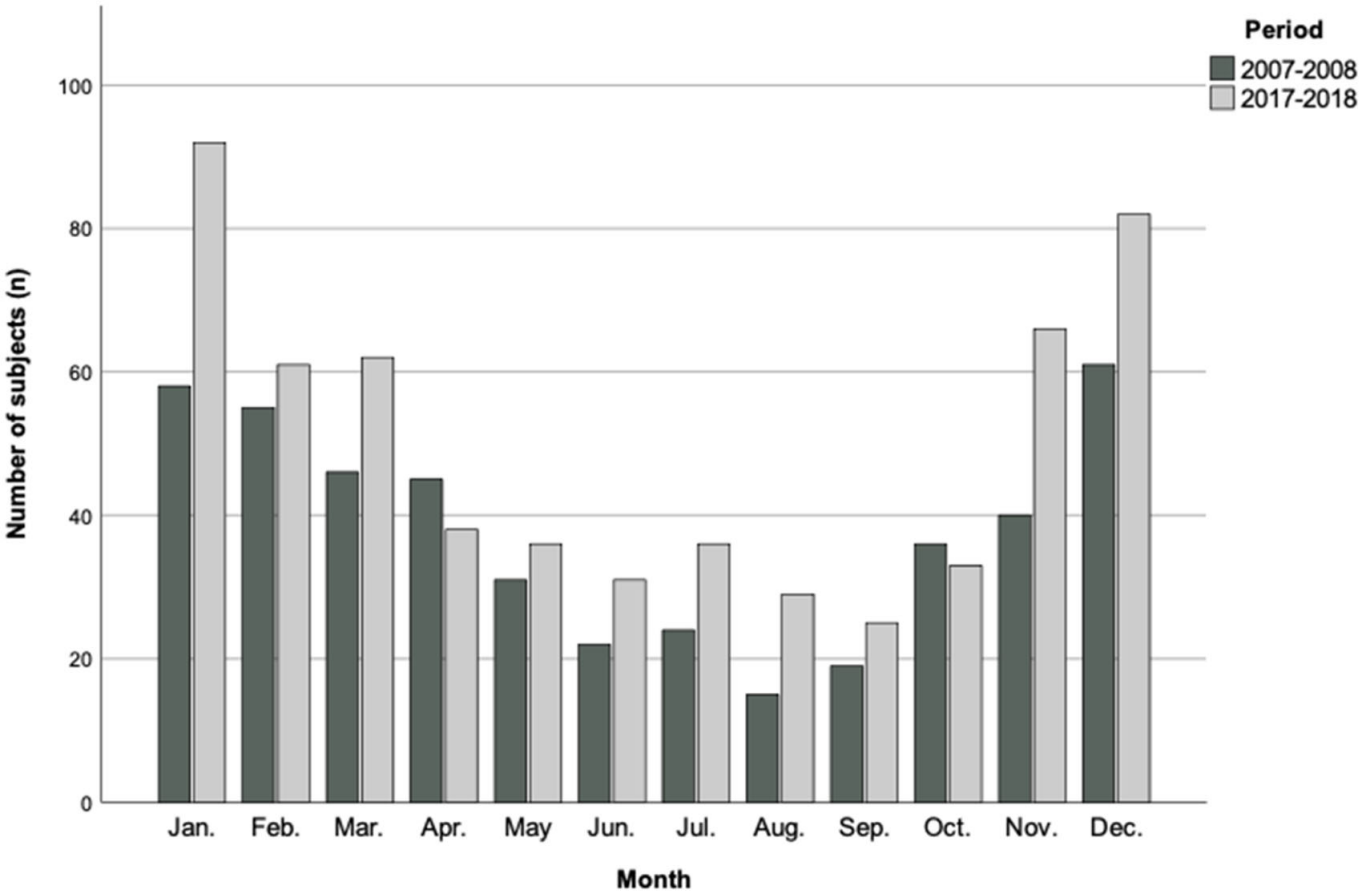
Monthly distribution of patients included in the two periods.

Overall, 1043 patients were included: 452 during the first period (2007–2008) and 591 during the second period (2017–2018). Monthly distribution of cases was similar in both periods (Figure 1). Six hundred and sixty-two (63.5) were men, and the mean age was 65 years (SD 18.8). In Table 1, we present the demographic characteristics and comorbid conditions of the study population. In the second period, patients were slightly older (63.4 vs 66.1 years; *p* = 0.02) and there was a higher proportion of women (33% vs 39.3%; *p* = 0.038). The proportion of patients with any underlying condition was similar, despite there were some differences in the type of comorbidities. A higher proportion of patients were vaccinated against influenza during the flu season in the second period.

**Table 1.** Basal characteristics and underlying conditions of patients included in the two study periods.

Characteristics	Total, *n* = 1043 (%)	2007–2008, *n* = 452 (%)	2017–2018, *n* = 591(%)	*p*
Mean (SD) age (years)	65.0 (18.8)	63.4(19.5)	66.14 (18.1)	0.02
Gender				
Male	662 (63.5)	303 (67)	359 (60.7)	0.038
Female	381 (36.5)	149 (33)	232 (39.3)	
Smoking (>5 cig/day)	229 (22)	108 (23.9)	121 (20.5)	0.200
Alcohol (>60 gr/d)	84 (8.1)	39 (8.6)	45 (7.6)	0.567
Nursing home	56 (5.4)	21 (4.6)	35 (5.9)	0.407
Any chronic underlying condition^a^	576 (55.2)	250 (55.3)	326 (55.2)	1.00
Any immunosuppressive condition^b^	201 (19.3)	88 (19.5)	113 (19.1)	0.937
Previous vaccination				
Pneumococcal vaccination (previous 5 years)	110 (10.5)	46 (10.2)	64 (10.8)	0.761
Influenza vaccination	263 (25.2)	76 (16.8)	187 (31.6)	<0.01

Data are expressed as numbers and percentages unless otherwise indicated. SD: standard deviation; IQR: interquartile range; HIV: human immunodeficiency virus. ^a^Underlying conditions: heart disease, chronic lung disease, chronic renal disease, neurological disease (vascular or degenerative) or cirrhosis. ^b^Immunosupressive conditions: HIV infection, solid organ transplant, haematologic malignancies, solid organ neoplasm.

Table 2 shows the pathogens documented in both periods. Bacterial aetiology did not change over the 10-years period. The only difference observed was a slight but significant increase in *S. aureus* CAP in the second period (*p* = 0.026). Methicillin-resistant *S. aureus* (MRSA) represented 1 out of 4 *S. aureus* isolates in the first period and 4 out of 13 in the second period (25% vs 23.5%, *p* = 1.0). There was a decline in the proportion of bacteraemic CAP in the second period (14.8% vs 9.6%, *p* = 0.012).

**Table 2.** Viral and bacterial pathogens documented in both periods.

	Total, *n* = 1043 (%)	2007–2008, *n* = 452 (%)	2017–2018, *n* = 591 (%)	*p*
Bacteria documented				
*Streptococcus pneumoniae*	382 (36.6)	171 (37.8)	211 (35.7)	0.517
*Staphylococcus aureus*	21 (2.0)	4 (0.9)	17 (2.9)	0.026
*Legionella pneumophila*	31 (3.0)	15 (3.3)	16 (2.7)	0.585
Other atypical bacteria	15 (1.4)	7 (1.5)	8 (1.4)	0.799
*Haemophilus influenzae*	28 (2.7)	13 (2.9)	15 (2.5)	0.847
*Pseudomonas aeruginosa*	17 (1.6)	11 (2.4)	6 (1.0)	0.086
Other bacteria	36 (3.5)	16 (3.5)	20 (3.4)	1.00
More than one bacterium	39 (3.7)	10 (2.2)	29 (4.9)	0.031
No bacteria detected	474 (45.4)	205 (45.4)	269 (45.5)	1.00
Virus documented				
Influenza A	–	–	31 (5.2)	–
Influenza B	–	–	34 (5.8)	–
Respiratory syncytial virus	–	–	16 (2.7)	–
Rhinovirus	–	–	20 (3.4)	–
Adenovirus	–	–	3 (0.5)	–
Coronavirus (not SARS-CoV2)	–	–	9 (1.5)	–
Metapneumovirus	–	–	5 (0.8)	–
Parainfluenza virus	–	–	1 (0.2)	–
Any bacterial or viral pathogen	623 (59.7)	247 (54.6)	376 (63.6)	0.004
Positive blood cultures	124 (11.9)	67 (14.8)	57 (0.6)	0.12

Regarding severity and clinical presentation, CURB-65 and PSI scores were similar in both periods (Table 3). The incidence of septic complications (complicated pleural effusion and septic shock) declined in the second period (6.4% vs 3.6%, *p* = 0.004 and 15.5% vs 5.3%, *p* < 0.001, respectively). On the other hand, respiratory failure and ICU admission were similar in both periods. During the second period, hospitalisation length was shorter than in the first period.

**Table 3.** Clinical information of CAP in the two periods.

	Total, *n* = 1043 (%)	2007–2008, *n* = 452 (%)	2017–2018, *n* = 591 (%)	*p*
Bilateral consolidation	131 (12.6)	59 (13.1)	72 (12.2)	0.706
Complicated pleural effusion^a^	50 (4.8)	29 (6.4)	21 (3.6)	0.04
Severity scores				
PSI high mortality classes (IV or V)	580 (55.7)	255 (56.7)	325 (55)	0.615
CURB-65 high mortality risk group (≥3 points)	238 (22.8)	92 (20.4)	146 (24.7)	0.102
ICU admission	94 (9.0)	41 (9.1)	53 (9.0)	1.0
Septic shock^b^	107 (10.3)	70 (15.5)	37 (6.3)	<0.001
Respiratory failure^c^	454 (43.5)	196 (43.4)	258 (43.7)	0.95
Invasive mechanical ventilation	54 (5.2)	30 (6.6)	24 (4.1)	0.068
Mean of hospitalisation days (SD)	10.2 (12.9)	11.9 (14.0)	8.9 (11.8)	<0.001
Mortality				
Mortality during hospitalisation	83 (8.0)	41 (9.1)	42 (7.1)	0.251
48-h mortality	26 (2.5)	11 (2.4)	15 (2.5)	1.0
30-day mortality	74 (7.1)	40 (8.8)	34 (5.8)	0.067

Data are expressed as numbers and percentages unless otherwise indicated. ICU: intensive care unit, SD: standard deviation, PSI: pneumonia severity index. ^a^Complicated pleural effusion: pH < 7.2, low glucose levels or evidence of microorganism by culture or gram stain. ^b^Septic shock: need of vasoactive drugs. ^c^Respiratory failure: pO_2_ in arterial blood lower or equal to 60 mmHg or peripheral pulse-oximetry lower than 90%.

Regarding mortality, although there were no significant differences between the two periods, there was a slight trend to lower 30-day mortality in the second period (8.8% vs 5.8%, *p* = 0.067). In the multivariate analysis, the independent factors associated with mortality were age (OR 1.05; 95%CI 1.3–1.7), septic shock (OR 5.38; 95%CI 3.00–9.67) and respiratory failure (OR 3.97; 95%CI 2.14–7.35). In order to know the variables associated with mortality in each period we performed a mortality analysis in each period which is shown in Table 4. Age and septic shock were variables independently associated with mortality in both periods. Respiratory failure was a risk factor but only in the first period (OR 7.57; 95%CI 2.83–20.2). Finally, we observed that in the second period, in which the rate of influenza vaccination was significantly higher (Table 1), influenza vaccination was an independent protective factor against mortality (OR 0.15; 95%CI 0.05–0.44).

**Table 4.** 30-Day mortality analysis (univariate and multivariate) in the two periods.

		Univariate analysis			Multivariate analysis	
		Alive at 30 days, *n* = 412 (%)	Death at 30 days, *n* = 40 (%)	*p*	Odds ratio (95% CI)	*p*
First period (2007–2008)	Mean (SD) age (years)	62.2 (19.7)	73.4(15.2)	0.001	1.037 (1.01–1.06)	0.002
	Nursing home	15 (3.6)	6 (15)	0.007	–	–
	Any underlying condition	222 (53.9)	28 (70)	0.066	–	–
	Influenza vaccination	71 (17.2)	5 (12.5)	0.657	–	–
	Bilateral consolidation	110 (11.4)	21 (28.4)	<0.001	–	–
	Respiratory failure	161 (39.1)	35 (87.5)	<0.001	7.571 (2.83–20.2)	<0.001
	Septic shock	53 (12.9)	17 (42.5)	<0.001	3.735 (1.74–8.02)	0.001
	Positive blood cultures	56 (13.6)	11 (27.5)	0.032	–	–

		Alive at 30 days, *n* = 557 (%)	Death at 30 days, *n* = 34 (%)	*p*	Odds ratio (95% CI)	*p*
Second period (2017–2018)	Mean (SD) age (years)	65.3 (18.2)	79 (10.8)	<0.001	1.092 (1.05–1.13)	<0.001
	Nursing home	29 (5.2)	5 (17.6)	0.001	–	–
	Any underlying condition	298 (53.5)	28 (82.4)	0.001	–	–
	Influenza vaccination	183 (32.9)	4 (11.8)	0.012	0.146 (0.049–0.436)	0.001
	Bilateral consolidation	60 (10.8)	12 (35.3)	<0.001	–	–
	Respiratory failure	233 (41.8)	25 (73.5)	0.001	–	–
	Septic shock	28 (5.0)	9 (26.5)	<0.001	8.676 (3.3–22.83)	<0.001
	Positive blood cultures	49 (8.8)	8 (23.5)	0.011	–	–

SD: Standard deviation, CI: confidence interval. Septic shock: need of vasoactive drugs; Respiratory failure: pO_2_ in arterial blood lower or equal to 60 mmHg or peripheral pulse-oximetry lower than 90%.

## Discussion

The epidemics and the bacterial aetiology of CAP did not suffer a relevant clinical change along a 10-year period. Bacterial pathogens did not differ over the years, although there was a slight but significant increase in the presence of *S. aureus* during the second period. During that period, the prevalence of *S. aureus* CAP was 2.9% which is similar to that reported in other studies [2]. *Staphylococcus aureus* has classically been related with influenza infection and several studies have demonstrated an increase in *S. aureus* detection during the flu-season [2,14]. Microbiological procedures did not differ between the two periods except for the determination of respiratory viruses, which was implemented in the second period. Therefore, we do not have epidemiological data on viral infection during the first period and it is not possible to concrete if the slight increase in *S. aureus* detection in the second could be explained by a rise in influenza virus infections.

In the era of antibiotic resistance, antibiotic susceptibility should constantly be assessed. Studies performed in other settings show a high proportion of MRSA, reaching 40–60% of all *S. aureus* isolates [14,15]. In our study, CAP caused by MRSA represents less than 0.5%, with no changes between the two periods. Considering the stability in the proportion of MRSA detection, we consider that empirical treatment should not be modified to offer MRSA coverage in our setting.

Concerning clinical and severity outcomes, despite the population in both periods had similar rates of underlying conditions and initial severity scores (CURB-65 and PSI), in the second period there was a decline of septic complications such as septic shock and complicated pleural effusion. The proportion of patients with positive blood-cultures decreased as well. These results go in the opposite direction than previous studies, in which an increase of septic complications over the years was reported [12,16]. Pneumococcal serotypes may be related with these changes since they are one of the determinants of severity in patients with pneumococcal disease and therefore in patients with CAP. Over the years, changes in the serotype prevalence have led to changes in the clinical presentation and severity of subjects with CAP. The implementation of the 7-valent vaccine brought a decline in the number of hospitalisations for pneumonia in children and adults [17]. Despite this, in the years after its implementation, concerns raised regarding the increase of non-vaccine serotypes [16,18,19] which came along with an increase of suppurated complications such as empyema [20,21]. In our study, between the two study periods, 13-valent conjugate pneumococcal vaccine was implemented, which could explain the changes mentioned above. Clinical changes after the introduction of the 13-valent vaccine have barely been reported. Simonetti *et al* assessed clinical outcomes 3 years after its introduction and reported stability in the rates of septic shock in 2010–2014 [12]. In comparison, in our study we have observed a decrease in septic complications in the second period, which could be explained by changes in the population pattern of serotypes in adults due to the implementation of the 13-valent vaccine in children. Further studies with pneumococcal serotype determinations are needed to make more concise statements on this subject.

Finally, in the mortality analysis we found that factors independently associated with 30-day mortality were age and septic shock. These findings are expected since they represent risk factors for mortality in other diseases as well [22,23], and this fact has not changed in the 10-year period.

Interestingly, influenza vaccination was a protective factor against mortality in the second period, in which vaccination rates were significantly higher than in the first period. With the increase in vaccination rates, influenza vaccine became a protective factor of mortality. It is known that an increase in the population vaccination rates can imply a herd effect and promote community protection [24]. Our results are concordant with other studies in which influenza vaccine is a protective factor against in-hospital death [25]. Therefore, we support that influenza vaccination is an important tool to prevent mortality in patients with CAP.

Our study has some limitations. First, the retrospective nature of the study entails design limitations. Second, it presents data from only one centre, so it might not be representative of other geographical areas, where some factors could differ. Third, the study was performed before the emergence of the SARS-CoV-2 pandemic, so we have not addressed the interaction between bacterial pneumonia and SARS-CoV-2 infection. However, in contrast to what occurs in patients with severe influenza in which bacterial co-pathogens are commonly identified, the overall proportion of bacterial coinfection among patients with COVID-19 seems to be only 6.9–8% [26,27]. Finally, we do not have data on pneumococcal serotypes in patients with pneumococcal pneumonia so we cannot make concise statements on the effect of pneumococcal vaccination in the decrease of septic complications.

## Conclusion

We have not found relevant differences in the bacterial aetiology of CAP over this 10-year period besides a slight increase in *S. aureus* infections. Moreover, there has been a decline in septic complications of CAP such as septic shock, bacteraemia, and complicated pleural effusion. Influenza vaccination is an important strategy to decrease mortality in patients with CAP. Therefore, in our opinion, the current management for patients with CAP stated in recent guidelines remains adequate in our setting.

## Author contributions

*Conception and design*: J. Sellarès-Nadal, J Burgos, V. Falcó; *Analysis and interpretation of the data*: J. Sellarès-Nadal, R. Sordé, J Burgos, V. Falcó; *Drafting of the paper*: J. Sellarès-Nadal, J Burgos, V. Falcó; *Critical revision for intellectual content*; MT. Martin-Gómez, R. Sordé, A. Anton, D. Romero-Herrero, P. Bosch-Nicolau, A. Falcó-Roget, C. Kirkegaard, D. Rodríguez-Pardo, O. Len; *Final approval of the version to be published*: J. Sellarès-Nadal, J. Burgos MT. Martin-Gómez, R. Sordé, A. Anton D. Romero-Herrero, P. Bosch-Nicolau, A. Falcó-Roget, C. Kirkegaard, D. Rodríguez-Pardo, O. Len, V. Falcó.

## Data Availability

The authors confirm that the data supporting the findings of this study are available within the article.
